# KRAS Mutation Status as a Prognostic Marker and Predictor of Therapy Response in Colorectal Cancer: an NCDB Analysis

**DOI:** 10.1007/s12029-026-01439-5

**Published:** 2026-03-05

**Authors:** Mohyeddine El Sayed, Melhem El Harati, Miller W. Shealy, Sassine Youssef

**Affiliations:** 1 Hollings Cancer Center, Medical University of South Carolina, Charleston, SC USA; 2 Department of Pulmonary and Critical Care, Medical University of South Carolina, Charleston, SC USA; 3 Department of Surgery, Medical University of South Carolina, Charleston, SC USA

**Keywords:** Colorectal cancer, National Cancer Database (NCDB), Overall survival, KRAS mutation, Stage-specific survival, Treatment pathways

## Abstract

**Background:**

KRAS mutation is a prominent biomarker in colorectal cancer (CRC) and is associated with resistance to anti-EGFR therapy in metastatic disease. However, its prognostic value across stages and its association with survival within common treatment pathways remain incompletely defined in large real-world cohorts.

**Methods:**

We performed a retrospective cohort study using the National Cancer Database, identifying 52,534 patients with CRC diagnosed between 2010 and 2021 with documented KRAS mutation status. Demographic, clinicopathological, and treatment variables were analyzed. Overall survival (OS) was evaluated using Kaplan–Meier methods and multivariable Cox proportional hazards models, performed overall and stratified by AJCC stage. Exploratory analyses evaluated the association of KRAS status with OS within stage-specific treatment pathways.

**Results:**

KRAS-mutant tumors comprised 39.9% of the cohort and were more frequently right-sided and metastatic at presentation and were less often poorly differentiated. KRAS mutation was independently associated with worse OS in the overall cohort (HR 1.12). Stage-specific associations were modest but statistically significant in stage II (HR 1.14), stage III (HR 1.16), and stage IV disease (HR 1.07). In exploratory treatment-pathway analyses, KRAS mutation was associated with inferior OS in several common regimens (e.g., surgery + chemotherapy in stage III and stage IV).

**Conclusion:**

KRAS mutation independently predicts poorer survival in CRC, particularly in earlier stages, and influences outcomes under multiple therapeutic regimens. These findings underscore the importance of KRAS testing to optimize individualized treatment strategies and guide novel interventions targeting KRAS-mutant CRC.

## Introduction

Colorectal cancer (CRC) is the third most frequently diagnosed malignancy and the second leading cause of cancer-related deaths [1]. This disease is heterogenous characterized by numerous genetic alterations and multiple carcinogenic pathways [2]. Three primary pathways are recognized in the progression of normal colorectal epithelium to adenocarcinoma: chromosomal instability (CIN), microsatellite instability (MSI), and the CpG island methylator phenotype (CIMP) [3]. Collectively, these pathways involve approximately 90 distinct mutations identified in CRC to date [4]. This extensive genetic heterogeneity underlies the diverse tumor characteristics and clinical outcomes observed among patients, even within the same TNM stage [5]. Many of these mutations significantly influence prognosis and treatment response [6–8].

One of the most critical biomarkers in CRC is the Kristen rat sarcoma viral oncogene homolog (KRAS), which is mutated in approximately 30–50% of cases, primarily in codons 12 and 13 [9, 10]. KRAS, a member of the RAS proto-oncogene family, encodes a GTP/GDP-binding protein that, when mutated, drives tumors metastasis and confers resistance to certain therapies [11, 12]. KRAS mutation status is a well-established predictive biomarker for anti-EGFR therapy and has been associated with prognosis in CRC, particularly in advanced stages [13–16]. In recent study, Yuan et al. reported substantially worse survival among CRC patients harboring KRAS mutations [16]. Furthermore, KRAS mutations independently correlate with treatment response in CRC [17–19]. A meta-analysis by Therkildsen et al. highlights that KRAS-mutated metastatic CRC is predictive of poorer outcomes in patients receiving anti-epidermal growth factor receptor (EGFR) therapy [17].

Despite these findings, the prognostic significance of KRAS mutation status across different CRC stages remains controversial, with inconsistent results reported in the literature [20–25]. The American Joint Committee on Cancer (AJCC) has emphasized the integration of molecular features alongside TNM staging to improve prognostic accuracy and support individualized risk stratification [26]. Accordingly, this study uses a national database to evaluate the association between KRAS mutation status and overall survival across CRC stages and to explore its relationship with survival across commonly used treatment pathways.

## Methods

In this retrospective cohort study, data were obtained from the National Cancer Database (NCDB) Participant User File (PUF 2021), a comprehensive clinical oncology database encompassing patient demographics, tumor characteristics, therapeutic interventions, and outcomes from more than 1,500 cancer treatment facilities across the United States [27]. KRAS mutation status was incorporated into NCDB as a site-specific factor for colon cancer beginning in 2010 and was subsequently harmonized within updated staging and data collection frameworks in later AJCC manuals. As all patient information was fully deidentified, Institutional Review Board (IRB) approval was not required.

The study population comprised patients aged ≥ 20 years diagnosed with CRC between 2010 and 2021 who had documented disease stage and documented KRAS mutation status (wild-type vs. mutated). Diagnoses were identified using the International Classification of Diseases for Oncology (ICD-O) codes. Patients with multiple primary tumors, missing tumor grade, or incomplete data on race/ethnicity, follow-up duration, metastatic status, or treatment information were excluded. Because KRAS testing is not uniformly performed across all CRC patients and is more commonly obtained in advanced-stage or treatment-eligible populations, restricting analyses to patients with documented KRAS status may introduce selection bias and limit generalizability to the broader NCDB CRC population.

Variables of interest included demographic factors (e.g., sex, age, race/ethnicity) and tumor-related characteristics such as primary tumor site, tumor size, grade, metastatic status, lymph-vascular invasion, and Charlson-Deyo comorbidity index. Treatment variables included surgery, chemotherapy, immunotherapy, and radiotherapy.

Categorical variables were summarized as frequencies and percentages and compared using Pearson’s chi-square test. Overall survival (OS) was the primary outcome and was defined as time from diagnosis to death or last follow-up. OS was estimated using the Kaplan–Meier method, with differences assessed using the log-rank test. Multivariable Cox proportional hazards regression models were used to evaluate the association between KRAS mutation status and OS in the overall cohort and within stage–stratified subgroups, with hazard ratios (HRs) and 95% confidence intervals (CIs) reported.

Exploratory analyses further examined the association between KRAS mutation status and OS across common stage-specific treatment pathways. For these analyses, stage-stratified Cox models were fit within treatment combinations when sample size permitted; estimates from small subgroups were interpreted cautiously due to limited power and potential instability. Variables statistically significant in univariate analyses were included in multivariable models. All analyses were performed using IBM SPSS Statistics, version 30.0 (IBM Corp., Armonk, NY), and two-sided p-values < 0.05 were considered statistically significant.

## Results

**Fig. 1:**
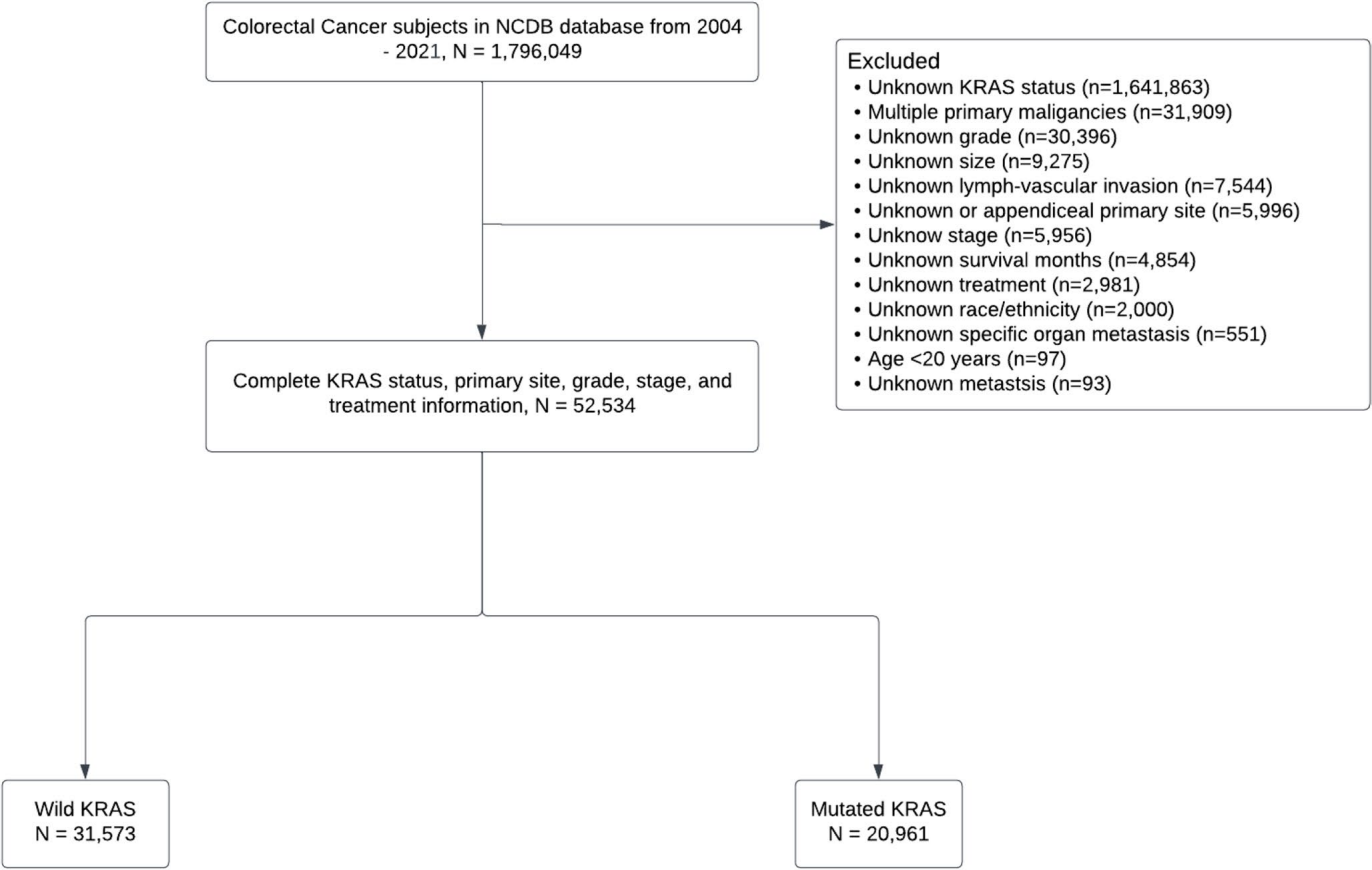
CONSORT diagram of included and excluded patients

The initial NCDB query identified 1,796,049 patients diagnosed with CRC between 2004 and 2021. After applying inclusion and exclusion criteria, the final analytic cohort consisted of 52,534 patients diagnosed between 2010 and 2021, of whom 60.1% harbored wild-type KRAS and 39.9% had KRAS mutations (Fig. 1). The distribution of KRAS mutation status differed across disease stages: wild-type KRAS was observed in 67.2% of stage 0/I cases, 63.7% of stage II, 61.9% of stage III, and 55.2% of stage IV, with the remaining proportions reflecting mutated KRAS.

Compared with patients who had wild-type KRAS, those with mutated KRAS included a higher proportion of females and non-Hispanic Black individuals. KRAS mutation was associated with right-sided primary site (55.3% vs. 46.1%; *P* < 0.001) and a higher proportion of stage IV disease at presentation (43.9% vs. 35.8%; *P* < 0.001). Patients with KRAS-mutant tumors presented with larger tumors and a higher incidence of distant metastasis, while rates of lymphovascular invasion were similar between groups. Surgical treatment was nearly universal across the cohort. Patients with KRAS-mutant tumors were more likely to receive chemotherapy and immunotherapy, whereas radiotherapy utilization did not differ significantly between groups. No clinically meaningful differences were observed in age or Charlson–Deyo comorbidity scores (Table 1).

**Table 1:** Patient demographics and clinical characteristics stratified by KRAS status

*N* = 52,534			
	Wild KRAS *N* = 31,573	Mutated KRAS *N* = 20,961	*P* value
Demographics			
Sex			
Male	52.6	50.4	**< 0.001**
Female	47.4	49.6	
Age group, years			
20–49	17.8	18.1	0.397
≥50	82.2	81.9	
Race/Ethnicity			
Non-Hispanic White	77.7	72.1	**< 0.001**
Non-Hispanic Black	11.1	16.3	
Hispanic	7.0	7.4	
Asian/Pacific Islander	3.8	3.7	
American Indian/Native	0.4	0.5	
Charlson-Deyo Score			
Score 0	72.3	72.2	0.775
Score 1	18.8	18.9	
Score 2	5.2	5.3	
Score 3+	3.7	3.6	
Tumor characteristics			
Primary site			
Right Colon	46.1	55.3	**< 0.001**
Left Colon	34.5	28.2	
Rectosigmoid Junction	9.0	6.9	
Rectum	10.4	9.6	
Grade			
well-differentiated	7.1	7.8	**< 0.001**
moderately differentiated	66.2	71.6	
poorly differentiated	22.7	17.7	
Undifferentiated	4.0	2.9	
Size, cm			
< 3.0	16.2	12.6	**< 0.001**
3.0–4.9	34.5	34.8	
≥ 5.0	49.3	52.6	
Stage			
0/I	9.9	7.3	**< 0.001**
II	20.0	17.1	
III	34.3	31.7	
IV	35.8	43.9	
Bone Metastasis	1.0	1.1	0.613
Brain Metastasis	0.2	0.3	0.152
Liver Metastasis	27.2	34.7	**< 0.001**
Lung Metastasis	5.0	9.5	**< 0.001**
Lymph-vascular Invasion	48.4	47.8	0.204
Treatments			
Surgery	99.6	99.6	0.867
Chemotherapy	65.5	70.4	**< 0.001**
Immunotherapy	13.3	15.6	**< 0.001**
Radiation	9.0	8.8	0.391

**Fig. 2:**
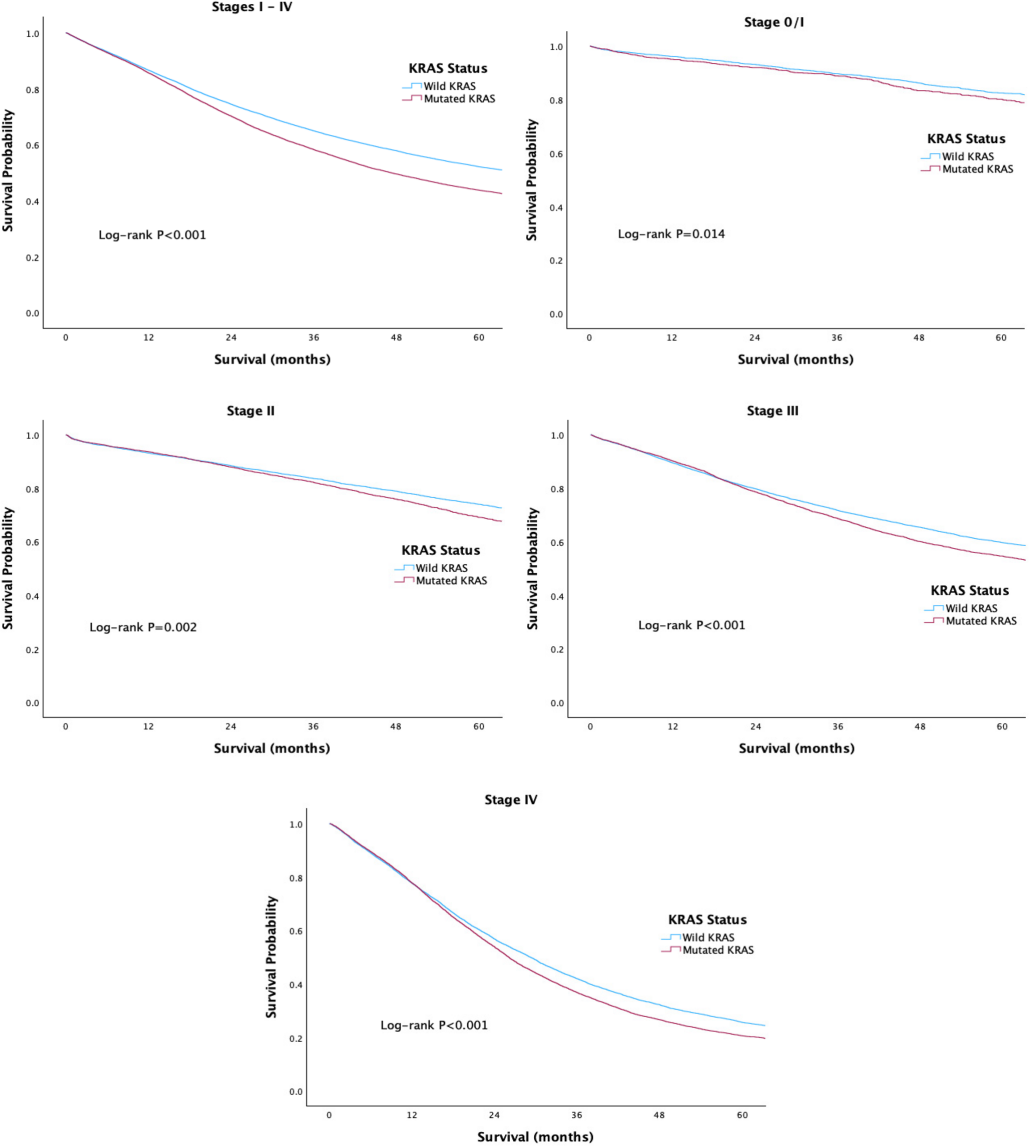
Overall survival of CRC patients stratified by KRAS status and tumor stage

Kaplan-Meier survival analysis revealed a significant association between KRAS mutation status and overall survival in CRC. Patients with wild-type KRAS tumors had superior 5-year overall survival compared with those harboring KRAS mutations (52.2% vs. 43.8%; *P* < 0.001). When stratified by disease stage, wild-type KRAS tumors were associated with improved overall survival across stage 0/I (82.4% vs. 80.1%; *P* = 0.014), stage II (74.0% vs. 69.1%; *P* = 0.002), stage III (59.7% vs. 54.6%; *P* < 0.001), and stage IV (25.7% vs. 20.6%; *P* < 0.001). Although survival declined with advancing stage regardless of KRAS status, KRAS-mutant tumors consistently demonstrated poorer outcomes (Fig. 2).

In multivariable Cox regression analyses, KRAS mutation was independently associated with worse overall survival across CRC stages. In the full cohort (stages 0–IV), KRAS-mutant tumors were associated with a 12% higher risk of mortality compared with wild-type disease (*P* < 0.001). The magnitude of this association varied by stage. KRAS mutation was significantly associated with increased mortality risk in stage II (HR = 1.14; *P* < 0.001), stage III (HR = 1.16; *P* < 0.001), and stage IV disease (HR = 1.07; *P* < 0.001). Notably, the relative increase in mortality risk was less pronounced in stage IV compared with stage II–III disease (Table 2).

**Table 2:** Overall survival by KRAS Status across CRC stages using multivariable cox regression

Stage	HR	95% CI	*P* value
Stages 0 – IV			
Wild KRAS	Referent	-	-
Mutated KRAS	1.12	1.09, 1.15	**< 0.001**
Stage 0/I			
Wild KRAS	Referent	-	-
Mutated KRAS	1.15	1.01, 1.31	0.040
Stage II			
Wild KRAS	Referent	-	-
Mutated KRAS	1.14	1.06, 1.23	**< 0.001**
Stage III			
Wild KRAS	Referent	-	-
Mutated KRAS	1.16	1.10, 1.21	**< 0.001**
Stage IV			
Wild KRAS	Referent	-	-
Mutated KRAS	1.07	1.04, 1.11	**< 0.001**

In stage-specific treatment pathway analyses, KRAS mutation was associated with worse overall survival across several common treatment combinations, although effect sizes varied. In stage II disease, KRAS mutation was associated with modestly increased mortality among patients treated with surgery alone (HR = 1.11; *P* = 0.029) and surgery plus chemotherapy (HR = 1.21; *P* = 0.033). Larger effect estimates were observed in the small subgroup receiving surgery and radiotherapy (HR = 4.56; *P* = 0.010). However, these estimates were based on limited sample sizes and should be interpreted cautiously. In stage III disease, KRAS mutation consistently predicted inferior survival among patients receiving surgery plus chemotherapy (HR = 1.15; *P* < 0.001) and surgery plus chemoradiation (HR = 1.31; *P* < 0.001), with similarly large but imprecise estimates in surgery-plus-radiation subgroups. In stage IV disease, KRAS mutation was associated with poorer survival among patients treated with surgery plus chemotherapy (HR = 1.07; *P* = 0.005) and surgery plus chemotherapy plus immunotherapy (HR = 1.15; *P* < 0.001) (Table 3).

**Table 3:** Impact of KRAS mutation on overall survival in CRC, stratified by disease stage and treatment combination

Treatment Combinations	HR	95% CI	*P* value	*N* (%)
Stage II				
Surgery only				6,738 (68.2)
Wild KRAS	Referent	-	-	4,442 (65.9)
Mutated KRAS	1.11	1.01, 1.22	**0.029**	2,296 (34.1)
Surgery & Chemotherapy				2,085 (21.2)
Wild KRAS	Referent	-	-	1,224 (58.7)
Mutated KRAS	1.21	1.02, 1.45	**0.033**	861 (41.3)
Surgery & Radiation				34 (0.3)
Wild KRAS	Referent	-	-	17 (50.0)
Mutated KRAS	4.56	1.43, 14.51	**0.010**	17 (50.0)
Surgery & Chemotherapy & Immunotherapy				108 (1.1)
Wild KRAS	Referent	-	-	64 (59.3)
Mutated KRAS	2.15	1.00, 4.63	0.050	44 (40.7)
Surgery & Chemotherapy & Radiation				883 (8.9)
Wild KRAS	Referent	-	-	529 (59.9)
Mutated KRAS	1.17	0.92, 1.48	0.192	354 (40.1)
Stage III				
Surgery only				3,249 (18.6)
Wild KRAS	Referent	-	-	1,986 (61.1)
Mutated KRAS	1.09	1.00, 1.19	0.062	1,263 (38.9)
Surgery & Chemotherapy				1,1673 (66.8)
Wild KRAS	Referent	-	-	7,271 (62.3)
Mutated KRAS	1.15	1.08, 1.22	**< 0.001**	4,402 (37.7)
Surgery & Radiation				44 (0.3)
Wild KRAS	Referent	-	-	27 (61.4)
Mutated KRAS	2.79	1.26, 6.13	**0.011**	17 (38.6)
Surgery & Chemotherapy & Immunotherapy				675 (3.9)
Wild KRAS	Referent	-	-	409 (60.6)
Mutated KRAS	1.09	0.89, 1.34	0.424	266 (39.4)
Surgery & Chemotherapy & Radiation				1,740 (10.0)
Wild KRAS	Referent	-	-	1,077 (61.9)
Mutated KRAS	1.31	1.14, 1.51	**< 0.001**	663 (38.1)
All 4				58 (0.3)
Wild KRAS	Referent	-	-	35 (60.3)
Mutated KRAS	0.73	0.38, 1.41	0.355	23 (39.7)
Stage IV				
Surgery only				2,615 (12.8)
Wild KRAS	Referent	-	-	1,444 (55.2)
Mutated KRAS	1.04	0.96, 1.14	0.320	1,171 (44.8)
Chemotherapy only				96 (0.5)
Wild KRAS	Referent	-	-	50 (52.1)
Mutated KRAS	1.19	0.73, 1.92	0.491	46 (47.9)
Surgery & Chemotherapy				10,213 (49.8)
Wild KRAS	Referent	-	-	5,565 (54.5)
Mutated KRAS	1.07	1.02, 1.12	**0.005**	4,648 (45.5)
Surgery & Immunotherapy				114 (0.6)
Wild KRAS	Referent	-	-	79 (69.3)
Mutated KRAS	0.90	0.49, 1.62	0.716	35 (30.7)
Surgery & Radiation				67 (0.3)
Wild KRAS	Referent	-	-	37 (55.2)
Mutated KRAS	0.81	0.35, 1.88	0.627	30 (44.8)
Chemotherapy & Immunotherapy				50 (0.2)
Wild KRAS	Referent	-	-	25 (50.0)
Mutated KRAS	0.72	0.37, 1.40	0.335	25 (50.0)
Surgery & Chemotherapy & Immunotherapy				5,932 (28.9)
Wild KRAS	Referent	-	-	3,275 (55.2)
Mutated KRAS	1.15	1.07, 1.22	**< 0.001**	2,657 (44.8)
Surgery & Chemotherapy & Radiation				920 (4.5)
Wild KRAS	Referent	-	-	529 (57.5)
Mutated KRAS	1.12	0.95, 1.31	0.173	391 (42.5)
All 4				434 (2.1)
Wild KRAS	Referent	-	-	255 (58.8)
Mutated KRAS	1.02	0.79, 1.32	0.886	179 (41.2)

## Discussion

In this NCDB cohort with documented KRAS status, KRAS mutation was independently associated with worse overall survival and showed differential associations with survival across several common treatment pathways. Within our cohort, 39.9% of patients had KRAS-mutant tumors, a rate that is consistent with other large studies [16, 20]. KRAS-mutant colorectal cancers exhibit a distinct clinicopathologic profile, characterized by lower grade, greater metastatic propensity, and a strong predilection for right-sided tumor origin, consistent with prior reports [28–31]. Gonsalves et al. demonstrated that KRAS-mutant tumors are 27% less likely to be poorly differentiated, while Gunal et al. reported that right-sided colon cancers are 150% more likely to harbor KRAS mutations [28, 29]. Together, these features underscore the unique biological behavior of KRAS-mutated tumors compared with their wild-type counterparts.

Our findings align with multiple prior studies demonstrating that KRAS mutation status is an independent prognostic factor in CRC [23, 25, 32]. Its negative prognostic impact is well established in stage IV disease, consistent with our results [15, 33, 34]. However, the influence of KRAS mutation in earlier-stage CRC has remained controversial. Several studies have reported no significant association between KRAS status and survival in stage II–III CRC [21, 22, 35]. In our analysis, KRAS mutation was not prognostic in stage 0/I disease but was associated with a modest (14–16%) increase in mortality risk in stage II–III CRC, consistent with recent evidence [13, 14, 25]. A meta-analysis by Formica et al. reported a 27% higher mortality risk in stage II–III KRAS-mutant tumors compared with wild-type counterparts, while Zhang et al. found mortality risk increases of 32% in stage I–II and 18% in stage III–IV CRC [14, 25]. Taken together, our findings suggest that the prognostic effect of KRAS mutation status diminishes in later disease stages, paralleling findings from Zhang et al. [25]. We attribute this phenomenon to the additional molecular events and the greater heterogeneity of advanced-stage CRC, which reduces the individual impact KRAS mutation status [36, 37].

To our knowledge, this is among the largest NCDB analyses evaluating KRAS status in relation to overall survival across stage-specific treatment pathways. However, because NCDB does not capture objective treatment response, recurrence, or progression, these findings should be interpreted as associations with overall survival rather than direct measures of treatment response. Accordingly, our results suggest differential survival outcomes by KRAS status across treatment pathways, rather than definitive evidence of treatment resistance or sensitivity.

Our results are partly supported by Deng et al., who evaluated the effect of KRAS mutation on adjuvant chemotherapy in 433 CRC patients. They reported that KRAS-mutant Stage II–III tumors were associated with worse outcomes after surgery alone, whereas no significant difference was observed among patient treated surgery plus chemotherapy [38]. Similarly, Ogino et al. found that KRAS mutation status did not substantially influence overall survival in stage III colon cancer patients treated with either 5-fluorouracil/leucovorin or a combination of irinotecan, 5-fluorouracil, and leucovorin [35]. Gavin et al. also reported that KRAS mutation status does not predict response to oxaliplatin in Stage II–III colon cancer [18].

We observed large effect estimates in small radiotherapy-containing subgroups; however, these strata included very few patients and yielded imprecise estimates with wide confidence intervals. As such, we interpret these findings as hypothesis-generating and not definitive evidence of differential radiosensitivity in KRAS-mutant CRC. While preclinical data suggest that oncogenic KRAS may contribute to radiotherapy resistance through mechanisms such as activation of the NRF2–53BP1 axis and enhanced non-homologous end-joining repair, dedicated clinical studies with detailed radiotherapy parameters are required to validate these mechanisms in patients [39].

Although anti-EGFR therapy has shown survival benefits in stage IV patients with wild-type KRAS, two clinical trials have shown no significant survival benefit from adding cetuximab in stage III colon cancer [40–42]. This discrepancy may be related to the association between high EGFR expression and more advanced T stage, greater lymph node involvement, and poorer differentiation, which may attenuate the benefit of anti-EGFR therapy in earlier-stage disease [43, 44].

In stage IV disease, our findings show that KRAS mutation is associated with poorer outcomes among patients treated with surgery and chemotherapy or surgery, chemotherapy, and immunotherapy. Several studies have highlighted the survival benefit of adding anti-EGFR therapy to chemotherapy in wild-type KRAS CRC, whereas this benefit does not extend to KRAS-mutant tumors [42, 45, 46]. A meta-analysis by Qiu et al. further confirmed that KRAS mutation status independently predicts treatment response to anti-EGFR therapy in metastatic CRC [47]. Additionally, bevacizumab, a monoclonal antibody against vascular endothelial growth factor (VEGF), has been shown efficacy in both wild-type and KRAS-mutant CRC, although a meta-analyses shown that wild-type KRAS patients derive a greater survival benefit than their KRAS-mutant counterparts [48, 49].

This study has several important limitations. First, KRAS status in NCDB is not uniformly tested across all CRC patients and is more commonly available in advanced-stage or treatment-eligible populations. Consequently, restricting analyses to patients with documented KRAS status may introduce selection bias and may limit generalizability to the broader NCDB CRC population, particularly earlier-stage disease. Second, KRAS was analyzed as a binary variable without codon- or exon-level detail (e.g., G12C, G12D, G13D), which may carry distinct prognostic implications. Third, treatment information in NCDB PUFs is limited to broad categories and lacks key details including chemotherapy regimen, biologic agents (e.g., anti-EGFR/anti-VEGF), dose intensity, treatment duration, radiotherapy dose/fields, sequencing, and completeness of systemic therapy; therefore, analyses across “treatment combinations” should be interpreted as associations rather than treatment-effect estimates. Fourth, several treatment strata—especially radiotherapy- and immunotherapy-containing groups—had small sample sizes, yielding unstable hazard ratio estimates and wide confidence intervals; these results are best considered exploratory. Fifth, the observational design is subject to residual confounding and treatment-selection bias that cannot be fully addressed with multivariable adjustment alone. Finally, NCDB captures overall survival but not cancer-specific survival, recurrence, progression, or cause of death.

## Conclusion

In this large NCDB cohort with documented KRAS status, KRAS mutation was associated with modestly worse overall survival across CRC stages, with the strongest relative association observed in stage II–III disease. KRAS-mutant tumors also demonstrated distinct clinicopathologic features, including a greater proportion of metastatic presentation. Because KRAS testing is not uniformly performed and NCDB treatment variables lack regimen- and agent-level granularity, these findings should be interpreted as prognostic associations rather than evidence of differential therapeutic response. Future studies using molecularly detailed, prospective datasets are warranted to clarify how KRAS variants refine risk stratification and guide treatment selection.

## Data Availability

The data that support the findings of this study were obtained from the National Cancer Database (NCDB). The NCDB is a joint project of the American College of Surgeons Commission on Cancer and the American Cancer Society. Access to the NCDB is restricted; researchers must apply for and obtain approval through the NCDB Participant User File (PUF) application process. As per NCDB data use agreements, the dataset cannot be shared publicly. Researchers interested in accessing the data may do so by submitting a data request directly to the NCDB at: https://www.facs.org/quality-programs/cancer/ncdb/puf/.
